# *De novo* assembly of the carrot mitochondrial genome using next generation sequencing of whole genomic DNA provides first evidence of DNA transfer into an angiosperm plastid genome

**DOI:** 10.1186/1471-2229-12-61

**Published:** 2012-05-01

**Authors:** Massimo Iorizzo, Douglas Senalik, Marek Szklarczyk, Dariusz Grzebelus, David Spooner, Philipp Simon

**Affiliations:** 1 Department of Horticulture, University of Wisconsin-Madison, 1575 Linden Drive, Madison, WI 53706, USA; 2 USDA-Agricultural Research Service, Vegetable Crops Research Unit, University of Wisconsin, 1575 Linden Drive, Madison, WI 53706, USA; 3 Department of Genetics, Plant Breeding and Seed Science, University of Agriculture Krakow, Al. 29 Listopada 54, 31-425, Krakow, Poland

**Keywords:** *de novo* assembly, Next generation sequencing, Mitochondrial genome, Plastid genome, Assembly structure visualization, *Daucus carota*, Intercompartmental DNA transfer

## Abstract

**Background:**

Sequence analysis of organelle genomes has revealed important aspects of plant cell evolution. The scope of this study was to develop an approach for *de novo* assembly of the carrot mitochondrial genome using next generation sequence data from total genomic DNA.

**Results:**

Sequencing data from a carrot 454 whole genome library were used to develop a *de novo* assembly of the mitochondrial genome. Development of a new bioinformatic tool allowed visualizing contig connections and elucidation of the *de novo* assembly. Southern hybridization demonstrated recombination across two large repeats. Genome annotation allowed identification of 44 protein coding genes, three rRNA and 17 tRNA. Identification of the plastid genome sequence allowed organelle genome comparison. Mitochondrial intergenic sequence analysis allowed detection of a fragment of DNA specific to the carrot plastid genome. PCR amplification and sequence analysis across different Apiaceae species revealed consistent conservation of this fragment in the mitochondrial genomes and an insertion in *Daucus* plastid genomes, giving evidence of a mitochondrial to plastid transfer of DNA. Sequence similarity with a retrotransposon element suggests a possibility that a transposon-like event transferred this sequence into the plastid genome.

**Conclusions:**

This study confirmed that whole genome sequencing is a practical approach for *de novo* assembly of higher plant mitochondrial genomes. In addition, a new aspect of intercompartmental genome interaction was reported providing the first evidence for DNA transfer into an angiosperm plastid genome. The approach used here could be used more broadly to sequence and assemble mitochondrial genomes of diverse species. This information will allow us to better understand intercompartmental interactions and cell evolution.

## Background

To date, 23 mitochondrial genomes in seed plants have been fully sequenced and analyzed http://www.ncbi.nlm.nih.gov/Genomes/. These mitochondrial genomes are extremely variable in size, ranging from 221 kb (*Brassica napus*) to 2,740 kb (*Cucumis melo*). Sequence analysis revealed that the most abundant portion of the mitochondrial genomes is non-coding [1], which includes “promiscuous” DNA of plastid and nuclear origin [2], as well as sequences of horizontal origin from foreign genomes [3-6]. Structural analysis, through use of Southern hybridization or paired-end data, revealed a high frequency of intra- and intermolecular recombination due to accumulation of repetitive sequences. This process has generated a structurally dynamic assemblage of genome configurations within a species [7-9] and a scrambling of gene order within closely related species [10]. This dynamic organization of the plant mitochondrial genome provides a powerful model for the study of genome structure and evolution. In addition, the increasing availability of plant organelle and nuclear genome sequence data provides an understanding of the mechanisms driving plant genome evolution. Indeed, there is a strong structural and functional interaction among plastid, mitochondrial, and nuclear genomes [11,12]. Transfer of DNA among these three compartments in higher plants has been reported, with exception of transfer into the plastid genome [13,14].

Despite its importance, technical obstacles of DNA isolation and sequence assembly limit the sequencing of mitochondrial genomes. Conventional approaches to mitochondrial genome sequencing involve extraction and enrichment of mitochondrial DNA, cloning, and sequencing. Large repeats and the dynamic mitochondrial genome organization complicate sequence assembly.

The development of next generation sequencing technologies (NGS), such as the Roche and Illumina platforms, provides a new opportunity for rapid characterization of mitochondrial genomes. Non-enriched whole genome DNA libraries, both shotgun and paired-end, include plastid and mitochondrial DNA that is sequenced along with the nuclear DNA during the sequencing run. NGS technologies have already been used for sequencing the small mitochondrial genome of nematodes [15,16], human [17] and fish [18] with no library enrichment. Recently, sequencing data from non-enriched libraries has been successfully used to assemble plastid genomes of wild and domesticated rice, mung bean, date palm, and milkweed [19-22]. The major limitations for use of this approach on *de-novo* assembly of mitochondrial genomes are the ability to overcome assembly problems related to large repeat regions, presence of promiscuous DNA, and sequence ambiguity due to sequencing technologies.

The aim of this study was to demonstrate how next generation sequence data from total genomic DNA can be used to *de-novo* assemble the mitochondrial genome of carrot (*Daucus carota*). In addition, intergenic sequence analysis provides evidence of a rare transfer of DNA into the plastid genome. This is the first report of mitochondrial genome transfer into an angiosperm plastid genome. The strategy used in this study has broad application to explore more mitochondrial genomes, to further investigate intra-cellular genome interaction and genome evolution.

## Results

### Assembly

Five different sets of 813,770, 814,668, 771,864, 704,918 and 692,688 454 shotgun reads (Table 1) with an average reads length ranging 354 to 419 nt, corresponding to an estimated nuclear genome coverage of 0.6×/set were used for initial assembly and 570,590 3 kb paired-end reads were used for connection verification. In addition, 50,598,879 Illumina reads of length 100 nt were used to correct homopolymer ambiguity. Each sequences set was independently assembled, each producing from 36,602 to 25,841 contigs (Table 1). Plastid assemblies resulted in five master circles with length ranging from 155,771 to 155,849 nt (Table 1). These five assemblies were then aligned with Kalign [23], generating a consensus sequence of 155,834 nt (Table 1). Alignment of the *de novo* assembly with the published carrot plastid genome [24] showed full-length coverage, with 99% identity relative to the published sequence including 49 nt of difference in cumulative sequence SNPs, and 433 nt of cumulative indels, with a maximum indel length of 20 nt.

**Table 1:** Summary of assemblies and consensus sequences of 454 whole genome sequences (WGS), plastid sequences (pt) and mitochondrial sequences (mt)

**Sequences dataset**	**# of reads**	**WGS assembly**	**pt assembly**		**mt assembly**	
		**# of contigs**	**# of contigs**	**Length (nt)**	**# of contigs**	**Length (nt)**
Set 1	813770	32215	11	155771	19	281052
Set 2	814668	36602	11	155845	19	281265
Set 3	771864	32963	15	155849	22	281164
Set 4	704918	27330	9	155820	22	281042
Set 5	692688	25841	12	155835	24	281242
Consensus				155834		281079

Mitochondrial assemblies were based on contig connections. In order to visualize these contig connections we developed and used bb.454contignet [http://www.vcru.wisc.edu/simonlab/sdata/software/]. The tool allows visualization of connections between gsAssembler contigs, along with contig size and average read coverage. This visualization allowed us to establish single and repeat contigs.

**Figure 1:**
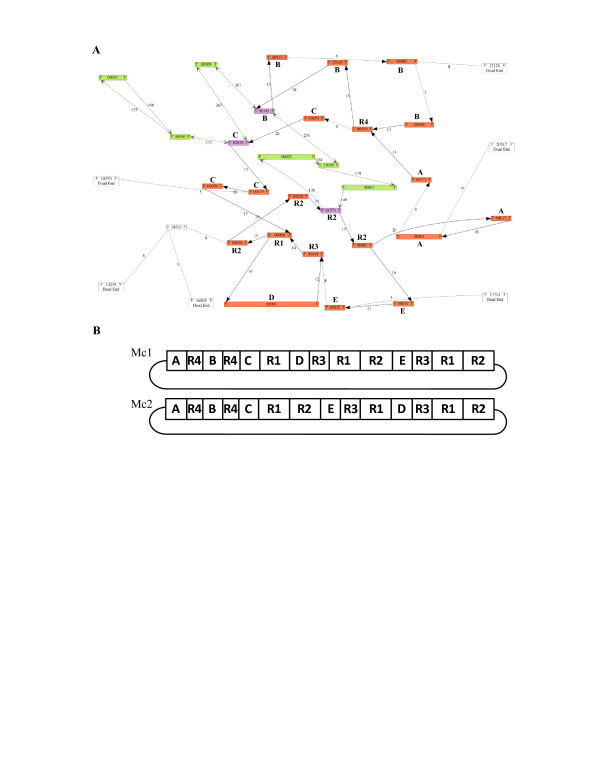
** Assembly strategy**. **A**: Visualization of contig connections of the *de novo* assembled carrot mitochondrial genome from sequence set 4, using bb.454contignet, including single (A, B, C, D, E) and repeated contigs (R1-R4). Numbers within boxes indicate contig ID as assigned by gsAssembler. The black arrows indicate the order of connections to concatenate the contig sequences into one of the two possible master circles; green arrows indicate the order of connections to concatenate the contig sequences from the plastid genome. Numbers between arrows indicate number of reads common to both contig ends. Boxes with different colors identify the origin of different contigs: red = mitochondrial genome; violet = contigs common to plastid and mitochondrial genome; green = plastid genome; white = putative nuclear genome connections. **B**: Schematic representation of the order of single copy regions (A, B, C, D and E) and repeated regions (R1-R4) in two possible master circles, Mc1 and Mc2.

**Figure 2:**
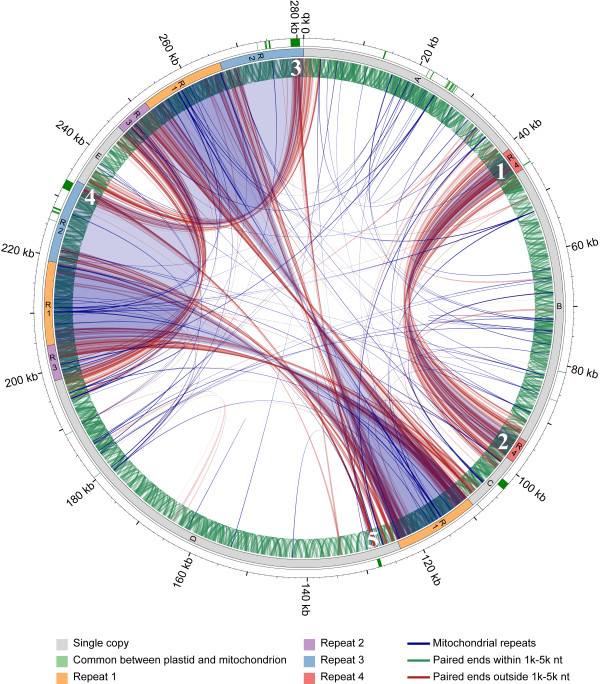
** Mapping of paired-end reads**. Results of mapping 3 kb 454 paired-end reads with both ends longer than 50 nt onto the *Daucus carota* mitochondrial genome. Green segments in the ideogram (outer circle) indicate sequences similar to the *Daucus carota* plastid genome; R1-R4 in the ideogram highlight large repeat regions; letters A, B, C, D, E indicate single copy regions; blue lines and shading indicate perfect and imperfect repeat sequences; green lines indicate reads that aligned at least once within a range of 1000 and 5000 nt of each other and were considered in agreement with the assembly; red lines indicate reads that mapped outside of this range (1000 to 5000 nt) and that were considered in disagreement with the assembly. Reads in disagreement with assembly were identified as reads that mapped within repeat regions (example 1, 2 connection, white numbers) or within mitochondrial-plastid similar regions that mapped within the expected range into the plastid genome and outside of the range into the mitochondrial genome (example 3, 4, 5 connections).

With 15 single copy and 7 repeated contigs from sequence set 4 the mitochondrial genome could be arranged in two possible master circles of 281,042 nt (Table 1, Figure 1, Additional file 1: Figure S1). Sequence analysis identified four large repeat regions, five single copy regions, and 12 possible connections between repeat sequences and flanking regions in the master circles. Sequence sets 1, 2, 3, and 5 had some small gaps in their assemblies, due to 3, 1, 1, and 3 missing connections, respectively (Additional file 2: Figure S2). Locations of these gaps were never shared between assemblies. Alignment of the five assemblies gave a complete consensus sequence of 281,079 nt (Table 1, Additional file 2: Figure S2). In order to confirm the 12 possible connections between repeat to repeat and repeat to single copy regions, we performed PCR and sequenced all possible amplicons spanning those regions. Sequence of these amplicons confirmed all 12 expected connections (Additional file 1: Figure S1). As a second verification, we mapped a set of 570,590 3 kb whole genome paired-end reads onto the mitochondrial assembly, with both ends ≥50 nt aligned. Mapped reads with ≥95% similarity and ≥85% of the length matching the assembled sequence, and reads that aligned at least once within a range of 1,000 to 5,000 nt of each other, were considered in agreement with the master circle assembly and 9,134 reads mapped at least once within this range covering the entire assembled genome (Figure 2, green lines). By contrast, reads appearing to be in disagreement (Figure 2, red lines) were alternative mappings near repeat region borders (Figure 2, examples 1, 2, white numbers) or with regions with plastid similarity that are within the expected range in the plastid genome and outside of the range in the mitochondrial genome (Figure 2, examples 3, 4, 5). These results confirmed repeat connections as well as contig connections.

In the case of sequences of ambiguous source, common to both mitochondrial and plastid genomes, we determined the true mitochondrial-version of these common sequences. We amplified and sequenced three regions with size of 1,298, 1,545 and 2,257 nt using the Sanger method. PCR results confirmed the size of the expected amplicons and these sequences were then incorporated into the mitochondrial assembly.

### Corrections and error rate

As 454 pyrosequencing is known to include insertion/deletion errors due to homopolymeric repeats [25], we corrected homopolymer regions of ≥ 5nt by mapping 51 million Illumina reads (corresponding to a 10x nuclear genome coverage) with quality ≥28, excluding the three regions common to plastid and mitochondrion, independently sequenced by the Sanger method (see above). In total 1,593 homopolymer sequences were detected, 23 in coding and 1,570 in non-coding regions (Table 2). After mapping, 40 errors were corrected, 6 in coding and 34 in non-coding regions, for a total length of 102 nt and an error rate of 1.14%.

**Table 2:** Illumina read mapping and corrections

**Sequence**	**Number of homopolymers**	**Cumulative homopolymers length (nt)**	**Number of errors**^**a**^	**Cumulative errors length (nt)**	**Percent of error**^**b**^
Non- coding	1570	8785	34	80	0.91
Coding	23	122	6	22	18.0
Total	1593	8907	40	102	1.14

Error rate estimation is for homopolymer sequences in coding and non-coding regions.

^a^ Corresponding to the number of corrected homopolymer sequences after mapping Illumina reads.

^b^ Calculated as cumulative ambiguity length/cumulative error length.

In addition to homopolymers, we also looked for ambiguity due to SNPs or single base insertions/deletions. Overall, no ambiguities were detected resulting in a final assembly of 281,132 nt.

### Coverage

Assemblies of mitochondrial and plastid genomes in this study were carried out using 454 sequence data from whole genome libraries (see Materials and Methods). In order to evaluate plastid and mitochondrial genome coverage obtained from all sets of shotgun 454 and Illumina reads used in this study, we mapped sequences against the assembled plastid and mitochondrial genomes using GNUMAP [26].

Mapping of five sets of 454 shotgun reads (813,770, 814,668, 771,864, 704,918 and 692,688 reads) gave a coverage ranging from 123× to 173× for plastid, and from 16× and 23× for mitochondrial genomes (Additional file 3, Table S1). Overall, 249,302 and 50,802 reads mapped in the plastid and mitochondrial genome suggesting that about 6.5% and 1.3% of the sequenced DNA was of plastid and mitochondrial origin, respectively.

For Illumina sequences, out of 51 million reads (corresponding to a 10× nuclear genome coverage), 6,949,870 sequences were successfully mapped to the plastid genome and 1,365,825 sequences were mapped to the mitochondria giving a 4,385x and 452x coverage, respectively (Additional file 3: Table S1). This suggests that about 13.7% and 2.7% of the sequenced DNA was of plastid and mitochondrial origin, respectively.

Analysis of GC content across the genomes indicate that in regions where the GC content is high the Illumina read coverage is reduced (Additional file 4: Figure S3 A1, A2). By contrast, high GC content did not affect 454 read coverage (Additional file 4: Figure S3 B1, B2).

These results confirmed that unenriched whole genome sequencing is a practical approach for *de novo* assembly of higher plant plastid and mitochondrial genomes.

### Southern blot analysis

**Figure 3:**
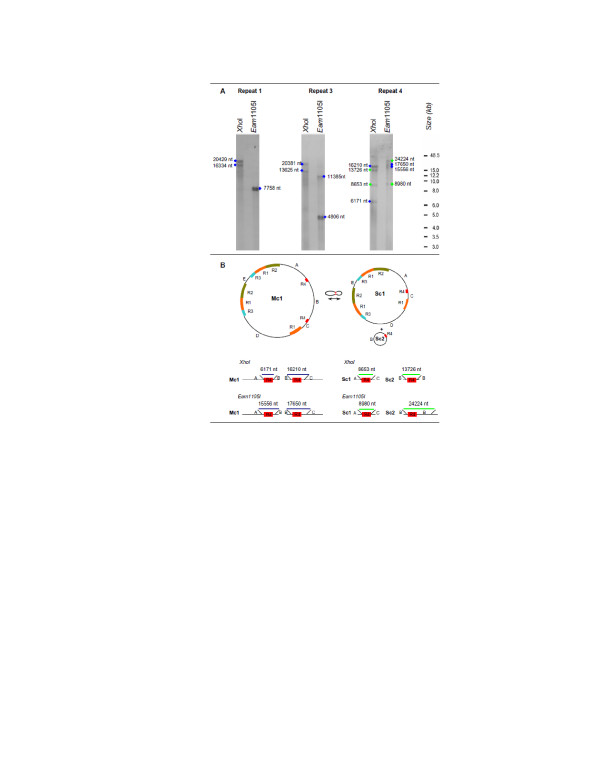
** Southern blot analysis. (A)**: Carrot DNA digested with *Xho*I or *Eam*1105I and hybridized with probes either within repeat 1 (R1), 3 (R3) or 4 (R4). **(B)**: Schematic representation of recombination across repeat 4 that underlies observed Southern hybridization results. Blue dots indicate expected size fragments corresponding to the Master circle 1 (Mc1), green dots indicate expected size fragments corresponding to the recombined Sub-circles (Sc) 1 and 2. Original picture of the gel including the size marker is reported in figure S4.

To evaluate the consistency of the assembly and possible recombination across repeats, a Southern blot experiment was carried out. Total genomic DNA was digested with two enzymes, *Xho*I and *Eam*1105I and hybridized with probes designed within repeats 1–3 and 4. Consistent with the assembled genome sequence, an expected hybridization pattern for both *Xho*I and *Eam*1105I was observed (Figure 3, A). In addition, the digestion pattern of repeat 4 with both *Xho*I and *Eam*1105I was consistent with the expected size fragments (8,653 nt and 13,726 nt for *Xho*I and 8,980 nt and 24,224 nt for *Eam*1105 I) of the recombinant sub-circles 1 and 2 (Figure 3B, Additional file 5: Figure S4).

### Genome size and content

**Figure 4:**
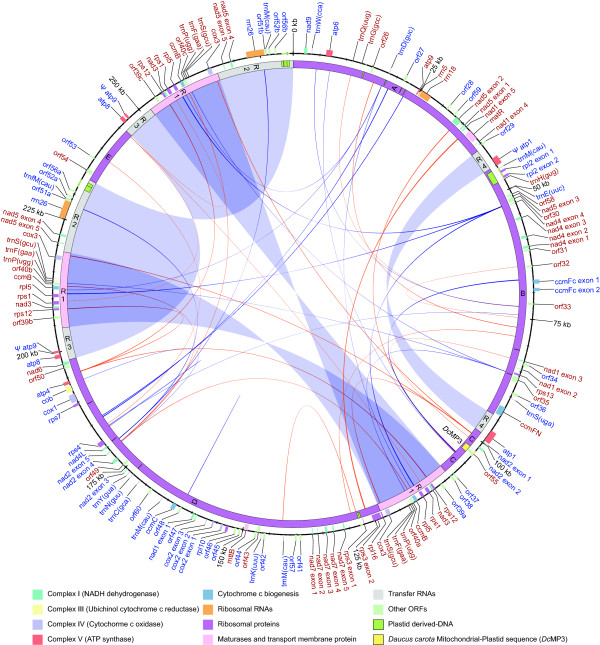
** Mitochondrial genome map.** Map of genes in the *Daucus carota* mitochondrial genome (281132 nt). It represents one of the two possible master circles, Mc1 (see Figure1B). Blue lines indicate direct repeats, red lines indicate inverted repeats. The genes with blue text color outside of the circle are transcribed in clockwise direction; those with red text color outside the circle are transcribed in counter clockwise direction. The inside circle indicates single copy regions A, B, C, D, E and repeated regions R1-R4. ψ indicates pseudogenes.

The 281,132 nt mitochondrial genome presented in Figure 4 is one of the two possible master circle conformations. After *Brassica napus* (221,853 nt) and *Silene latifolia* (253,413 nt), the carrot mitochondrial genome is one of the smallest mitochondrial genomes sequenced to date among the angiosperms. The overall GC content of carrot (45.4%) is comparable to other angiosperms [27,28] (Table 3). Considering alignments with minimum length of 50 nt, the portion of the genome exhibiting 80% or more similarity with *Nicotiana, Vitis* and *Carica* constitutes 47.6%, 45.0% and 44.5%, respectively. Intergenic spacer regions represent the largest part of the genome with 224,526 nt (79.9%). Coding-exons represent 16.2% (46,063 nt) of the genome, and RNA-coding genes constitute 3.7% (10,547 nt), with rRNA accounting for 3.0% (8,565 nt) and tRNA accounting for 0.7% (1,982 nt) of the genome (Table 3).

**Table 3:** Summary of mitochondrial genome content

**Feature**	**Length (nt)**	**Percent**
GC content	-	45.4
Coding exons	46063	16.4
tRNA coding genes	1982	0.7
rRNA coding genes	8565	3
Intergenic sequences	224526	79.9
Chloroplast like	5701	2

A BLAST search against a local mitochondrial protein and nucleotide database identified 44 putatively functional protein-coding genes and 3 genes coding for ribosomal RNAs (Additional file 6: Table S2). For all of these genes, open reading frames (ORFs) were detected, confirming the quality of the consensus sequence. Distribution of genes is illustrated in Figure 4. As in other angiosperm mitochondrial genomes, most of the known genes encode for proteins of the electron transport chain, with 9 subunits of complex I (*nad12344 L567* and *9*), one subunit of complex III (*cob*), three subunits of complex IV (*cox12* and *3*), five subunits of complex V (*atp1468* and *9*) and four genes involved in the biogenesis of cytochrome c (*ccmBccmCccmFc* and *ccmFn*). Truncated copies of *atp1* and *atp9* were detected, confirming observations previously reported by [29,30]. In addition, two conserved ORFs, coding for a maturase (*matR*) mapped within the *nad1* intron and another ORF named *mttB* coding for a transporter protein, were detected.

The number of ribosomal and succinate dehydrogenase proteins is usually variable among different species (Additional file 6: Table S2). Annotation of the carrot mitochondrial genome allowed identification of 9 putatively functional genes coding for ribosomal proteins (*rpl5*, *rpl10*, *rpl16*, *rps1*, *rps3*, *rps4*, *rps7*, *rps12* and *rps13*) of mitochondrial origin and one, *rpl2* of plastid origin. The genome appears to lack functional copies of the succinate dehydrogenase genes *sdh3* and *sdh4*.

Because they are in repeat regions, one gene (*atp8*) is duplicated, and six genes occur in triplicate (*ccmB*, *cox3*, *nad3*, *rpl5*, *rps1* and *rps12*). Detection of ORFs allowed identification of 19 group II introns, 7 of which are trans-spliced.

BLAST searches against a local nucleotide mitochondrial database and detection with tRNA scan-SE allowed identification of genes coding for 18 tRNAs (Table 4). One is a pseudogene, coding for a plastid-derived tRNA-Ile (UAU) with a deletion in the coding sequence. Out of the 17 tRNAs, four are of plastid origin and 13 of mitochondrial origin. These tRNA genes recognize 15 amino acids (*Phe*, *Met*, *Ser*, *Pro*, *Tyr*, *His*, *Gln*, *Asn*, *Lys*, *Asp*, *Glu*, *Cys*, *Trp*, *Ser* and *Gly*). Thus, tRNA genes for six amino acids are missing in the carrot mitochondrial genome.

**Table 4:** tRNA content of carrot mitochondrial genome

**Anti- codon**	**Amino acid**	**tRNA gene**	**Anti- codon**	**Amino acid**	**tRNA gene**	**Anti- codon**	**Amino acid**	**tRNA gene**
UUU	Phe	-^a^	UCU	Ser	-	UAU	Tyr	-
UUC	Phe	**trnF(gaa)*x3***^b^	UCC	Ser	-	UAC	Tyr	**trnY(gua)**
UUA	Leu	-	UCA	Ser	**trnS(uga)**	UAA	-	-
UUG	Leu	-	UCG	Ser	-	UAG	-	-
CUU	Leu	-	CCU	Pro	-	CAU	His	
CUC	Leu	-	CCC	Pro	-	CAC	His	*trnH(gug)*
CUA	Leu	-	CCA	Pro	**trnP(ugg)*x3***	CAA	Gln	**trnQ(uug)**
CUG	Leu	-	CCG	Pro	-	CAG	Gln	-
AUU	Ile	-	ACU	Thr	-	AAU	Asn	-
AUC	Ile	-	ACC	Thr	-	AAC	Asn	*trnN(guu)*
AUA	Ile	*trnI(uau)ψ*^c^	ACA	Thr	-	AAA	Lys	**trnK(uuu)**
AUG	Met	**trnfM(cau)*x2***, **trnM (cau)*x2***, *trnM(cau)*^d^	ACG	Thr	-	AAG	Lys	-
GUU	Val	-	GCU	Ala	-	GAU	Asp	-
GUC	Val	-	GCC	Ala	-	GAC	Asp	*trnD(guc)*
GUA	Val	-	GCA	Ala	-	GAA	Glu	**trnE(uuc)**
GUG	Val	-	GCG	Ala	-	GAG	Glu	-
UGU	Cys	-	AGU	Ser	-	CGA	Arg	-
UGC	Cys	**trnC(gca)**	AGC	Ser	**trnS(gcu)*x3***	CGG	Arg	-
UGA	-	-	AGA	Arg	-	GGA	Gly	-
UGG	Trp	**trnW(cca)**	AGG	Arg	-	GGG	Gly	-
CGU	Arg	-	GGU	Gly	-			
CGC	Arg	-	GGC	Gly	**trnG(gcc)**			

^a^ Missing;

^b^ Bold indicates tRNA of mitochondrial origin;

^c^ ψ: pseudogene;

^d^ Italic indicate tRNA of plastid origin.

In order to investigate the presence of the seven missing mitochondrial genes (*sdh3*, *sdh4*, *rpl2*, *rps2*, *rps10*, *rps14* and *rps19*) in the nuclear genome we aligned the corresponding sequences from the mitochondrial genome of the species most closely related to carrot (see Materials and Methods) against a local database containing all the assembled sequences and raw reads used in this study. For three genes, *sdh3*, *rps10* and *rps14*, a corresponding contig with conserved ORFs was detected in the nuclear genome (data not shown). None or insignificant matches were identified for the remaining four genes, *sdh4*, *rpl2*, *rps2* and *rps19* suggesting that these genes were not present in this dataset.

### Intergenic sequences

After annotation of conserved genes, we searched for other ORFs and plastid regions in the intergenic sequences and ORFs longer than 300 nt were annotated (Figure 4, *orf*26–60). In total, 35 ORFs ranging from 300 to 1,122 nt in length were detected (Figure 4). Out of these, 3 ORFs were conserved among mitochondrial genomes, with the best match of *orf*58 to *orf*147 of *Nicotiana tabacum*, of *orf59* to *orf*115b of *Brassica napus* and of *orf60* to *orf*122 of *Beta vulgaris*. Out of the remaining ORFs, 28 (*orf*26–53) had unknown function, and 4 (*orf* 54–57) were similar to truncated copies of hypothetical proteins from *Vitis vinifera* and *Arabidopsis thaliana* nuclear genes.

BLAST analysis of intergenic sequences against a local database containing all published plant plastid genomes allowed detection of 16 fragments with similarity to plastid sequences, with length ranging from 45 to 1,298 nt, accounting for 5,701 nt (2.0% of the genome) (Figure 4, green box).

### Repeats

With a total of 74 repeats ranging from 37 to 14,749 nt, the carrot mitochondrial genome has the lowest number of repeats among the sequenced plant mitochondrial genomes (Table 5, Figure 4, red and blue line). All but one are dispersed repeats. Most of the repeats (about 90%) are between 20 and 202 nt in length accounting for just 2.0% of the total genome coverage. Nine large repeats ranging from 4,220 to 14,749 nt account for 44.0% of the genome. Analysis of repeat orientation allowed the detection of 52 direct repeats (Figure 4, blue line) and 22 inverted repeats (Figure 4, red line). The insertion of the large repeat 1, between repeat 2 and 3, forms a 35 kb super-repeat. After wild cabbage [8], this is the largest repeat region described in eudicot mitochondrial genomes to date.

**Table 5:** Distribution of repeats in the carrot mitochondrial genome

**Repeat length (nt)**	**Number of repeats**	**Genome coverage (%)**	**Orientation**	
			**Direct**	**Inverted**
20–40	10	0.14	9	1
41–60	18	0.34	10	8
61–80	6	0.14	4	2
81–100	6	0.19	3	3
101–202	25	1.18	17	8
4220–14658	9	44	9	0
Total	74	45.99	52	22

### Evidence of DNA transfer into the plastid genome

**Figure 5:**
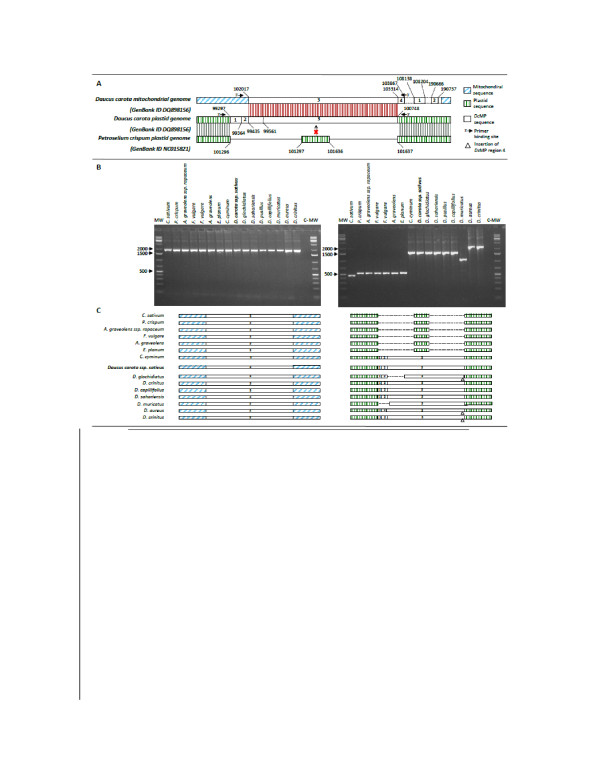
** DNA insertion in plastid genome.** Verification of DNA insertion into the *Daucus carota* plastid genome. **A**: Comparison of *Dc*MP regions 1, 2, 3 between *Daucus carota* plastid and mitochondrial genomes and *Petroselinum crispum* plastid genome; red shading indicates similarity between mitochondrial and plastid genome, gray shading indicates similarity between plastid genomes; **B**: PCR amplification with primers specific for mitochondrial *Dc*MP region 3 (left) and plastid *Dc*MP regions 1, 2, 3 (right) in different Apiaceae species; **C**: Comparison of mitochondrial *Dc*MP region 3 (left) and plastid *Dc*MP regions 1, 2, 3 (right) sequences across several Apiaceae species.

Recently Goremykin and colleagues [5], while analyzing the *Vitis vinifera* mitochondrial genome, detected two sequences of 74 (Figure 5, region 2) and 126 nt (Figure 5, the left terminal part of region 3) which were similar to the carrot plastid genome (positions 99,364-99435 and 99,437-99561 of GenBank: NC 008325). Both sequences are contained in a 1,452 nt carrot plastid sequence (position 99,297-100,748) that is absent in other published plastid genomes. BLAST analysis revealed that the 74 nt sequence is similar to *cox1* gene, suggesting a transfer of DNA from the mitochondrial to the plastid genome. Given this unexpected finding and no experimental confirmation, the authors concluded that an evaluation of this region of the carrot plastid assembly would be needed. In order to refer to this sequence we coded it as *Daucus carota* Mitochondrial Plastid sequence (*Dc*MP).

Here we confirm the presence of this region in the carrot plastid genome in the inbred carrot line B493B analyzed in this study (Figure 5A*Dc*MP region 1, 2, 3). In addition, analysis of intergenic sequences of the carrot mitochondrial genome revealed that this entire sequence is present in the mitochondrial genome as three separate pieces. Comparison of *D. carota* to *Petroselium crispum* [GenBank: NC 015821] revealed that the carrot plastid genome lacks a 339 nt sequence (Figure 5A, position 101,297-101,636 of *P. crispum* plastid genome) present in the *P. crispum* and other more taxonomically distant species because of the replacement with the *Dc*MP region.

In order to clarify the origin of this region, we first designed primers specific of flanking regions 1, 2 and 3 of the plastid genome or region 3 of the mitochondrial genome. We then amplified and sequenced these regions in different Apiaceae species. PCR results revealed a consistent conservation of the region 3 in the mitochondrial genomes; in fact an expected amplicon of 1,935 nt was amplified across all species (Figure 5B, left). By contrast, a polymorphic pattern has been obtained when the plastid flanking sequences were used to anchor the primers (Figure 5B, right). A short fragment of 500 nt was amplified in all non *Daucus* species including parsley (*Petroselium crispum*), fennel (*Foeniculum vulgare*), celery (*Apium graveolens*), plains eryngo (*Eryngium planum*) and 400 nt in coriander (*Coriandrum sativum*). The only exception was for cumin (*Cuminum cyminum)* which has the same pattern as carrot. With the exception of *D. aureus*, *D. crinitus* and *D. muricatus,* where amplicons of about 2,000, 2,000 and 1,100 nt were obtained, respectively, other *Daucus* species and cumin produced the amplicon of expected size, 1,618 nt.

Sanger sequence of above described PCR products confirmed the presence of the large region 3 in the mitochondrial genome of all *Daucus* and non *Daucus* species. It also confirmed, the absence of the insertion of regions 1, 2 and 3 in the plastid genome of non *Daucus* species (Figure 5C). These sequences and its taxonomic distribution in *Daucus* and close relative cumin [64; see Discussion] provide strong evidence of a transfer of DNA into the plastome. In addition a few variants were observed. An insertion observed in the plastid *Dc*MP region of wild species *D. aureus*, *D. crinitus* and *D. glochidiatus* is similar to a mitochondrial sequence contiguous to the *Dc*MP region 3 (Figure 5 A and C, region 4). This suggests that this region now identified as region 4 was part of the original insertion in the plastid genome of the ancestral progenitor of carrot. Sequence analyses of the plastid *D. murucatus Dc*MP region revealed that the shorter amplicon is due to a deletion of regions 1 and 2.

To investigate the sequence characteristics that might provide a possible explanation of this transfer in the carrot plastid genome, we aligned the plastid and mitochondrial sequences of regions 1, 2 and 3 against NCBI nucleotide and protein databases. The alignment demonstrated that region 1 was an intergenic sequence with no similarity to any other nucleotide or protein sequence present in databases. As previously reported by Goremykin and colleagues [5], region 2 is part of the *cox1* gene. It is more interesting that blastx alignment of region 3 revealed multiple matches with nuclear retrotransposable elements from different species including *Vitis vinifera* [GenBank: CAN77047 and CAN79321] and *Populus trichocarpa* [GenBank: XP 002329638 and XP 002334157] (Additional file 7: Figure S5).

## Discussion

### Implications for mitochondrial genome study

Next generation sequencing technologies have revolutionized molecular biology by making whole genome sequencing projects possible for any species. Low coverage whole genome shotgun sequencing has proven a valuable approach for rapid and easy acquisition of a large amount of sequence data at relatively low cost. Low coverage data is useful for marker acquisition as well as the assembly of plastid genomes [20,21,31-33]. Despite its power, few examples of the application of NGS on *de novo* assembly of plant mitochondrial genomes have been reported. Recently Straub and colleagues [22] attempted to assemble the milkweed mitochondrial genome using Illumina data from unenriched whole genome DNA. The assembly resulted in a partial mitochondrial genome assembly of 115 contigs. Here we demonstrate how 454 data from whole genome DNA library can be used for a complete *de novo* assembly of the mitochondrial genome of *Daucus carota.* Adequate coverage, sufficient read length, and application of a bioinformatics analysis using a tool such as bb.454contignet were crucial for the final assembly.

Our sequencing library was prepared from whole genome DNA. The fraction of reads of plastid and mitochondrial genome origin obtained from such a library can vary depending on the age of tissue, DNA extraction method, relative size of the organelle and nuclear genomes, and species [34]. Adequate coverage is always necessary for a complete plastid and mitochondrial genome assembly, but this coverage likely can be obtained, even with a low nuclear genome coverage. The fact that we were near the lower limit can be seen from a comparison of the five assembly replicates, which suggests that if the coverage is too low, the complete assembly of the mitochondrial genome can begin to fail apparently due to isolated gaps in the sequence due to random chance.

In our study, leaf tissue was collected from plants without any pretreatment (such as darkness) that could reduce the amount of plastid or mitochondrial DNA. The same DNA extraction was sequenced by both platforms. We observed approximately twice the organellar fraction from Illumina sequencing, relative to 454. The interpretation of this observation is difficult as many factors could be involved. Here we observed that Illumina is more sensitive to regions with variable GC content than is 454. In a range of GC content of 20–50% other authors reported a positive correlation between GC contents and sequence coverage [35,36]. Since organellar DNA is typically higher in GC content than nuclear DNA, we suggest this as one of possible cause of the higher representation of organellar fraction from Illumina sequencing.

Development of new bioinformatic tools is often a crucial point for interpreting DNA/RNA sequence information into biological information [37]. Repeat regions are the primary difficulty in genome assembly, and the length of reads relative to the length of repeats is a key factor in the ability to assemble a genome [38]. Reads from repeats longer than the read length could assemble to multiple possible sequences, some of which may not exist *in vivo*. These cannot be collapsed into a single unambiguous sequence, thereby causing breaks in contigs, where one end of a contig can then lead to two or more adjacent contigs which continue the sequence. Some assemblers such as gsAssembler make this connection information available. We developed and used bb.454contignet http://www.vcru.wisc.edu/simonlab/sdata/software/ to parse, filter, and then graphically visualize these contig connections. This tool was very useful for our small-scale assembly project, and it could be suitable for other applications, such as assembly of Bacterial Artificial Chromosome sequences, or specific regions in a whole genome assembly. As confirmation of the validity of our procedure, another group has successfully assembled another genome using this tool [39]. A similar tool, ABySS-Explorer has been developed for assemblies generated by ABySS [40]. However, our results demonstrate the utility of shotgun 454 sequences with a size of 300–500 nt, making the experiment cost efficient, and we confirmed the assembly by PCR, Southern blot and mapping 3 kb paired end data.

Full *de novo* assembly of the mitochondrial genome can be very difficult due to repeat regions as well as its dynamic organization due to frequent recombination events. Recent advances in Illumina and Roche 454 sequencing technologies have allowed increasing read length up to 150 and 1000 nt, respectively [http://www.illumina.com, http://my454.com]. In addition, both NGS technologies officially support paired sequence data of up to 5 kb for Illumina platform and up 20 kb for Roche 454 platform. Comparison of the distribution of the number and the length of the repeat sequences among sequenced mitochondrial genomes and the whole range of longest sequencing data achievable by both technologies demonstrates the power of NGS to overcome most of the assembly ambiguities due to repeats across these genomes. Moreover, use of long shotgun sequences alone, such as 454 reads, would resolve assembly ambiguity across a large portion of repeat regions detected among these mitochondrial genomes (Additional file 8: Figure S6). By contrast, use of short shotgun reads such as Illumina could still result in a high number of contigs and connections that could make the assembly unreliable. Paired sequence data can support and confirm assembly, and supply information about structural variants due to recombination across repeats. The ongoing development of third generation sequencing technologies such as PacBio [http://www.pacificbiosciences.com/], able to produce longer reads, will facilitate sequencing of new organelle genomes.

The usefulness of new organelle genome sequences will be reduced if their quality is reduced by sequence errors. Next generation sequencing technology is known to generate a sequence error rate higher than the traditional Sanger method [41]. Most of these errors can be corrected with high sequence coverage. However, errors due to inaccurate determination of homopolymer length, particularly when ≥5 nt, are characteristic of pyrosequencing (Roche 454), and are not resolvable by higher coverage. These errors can result in an inaccurate sequence annotation if the error occurs in a reading frame. In our study, homopolymer ambiguity was detected in both coding and non coding sequences. Availability of Illumina sequences allowed us to correct these homopolymer errors as described above. After corrections, ORF detection confirmed contiguous reading frames of all of the genes predicted by BLAST alignment, an additional verification of the correctness of the finished sequences. Combining these two technologies for this type of correction has already been described [42]. The high number (1,593) of homopolymer regions observed in this study would make verification of every homopolymer region by Sanger sequencing uneconomical. By contrast, the low number of ambiguities in coding regions [23] in such a small scale sequencing project would allow the use of a Sanger sequencing approach as an alternative cost-efficient way to correct ambiguity in those regions, and insure correct annotation of genes.

### Genome organization

Carrot is the first mitochondrial genome sequenced among the large euasterid II clade, and only the second, along with *N. tabacum*, among asterids. The size of the genome (281,132 nt) is similar to a previous estimation (255,000 nt) made by Robinson and Wolyn [43] based on restriction digestion mapping. Sequence analysis revealed that the closest mitochondrial genome in terms of similarity was tobacco, confirming their phylogenetic relationship in the euasterid II clade. The gene content of the carrot mitochondrial genome confirms the previous report of Adams et al. [44] where the authors used Southern hybridization to survey mitochondrial gene presence or loss across 280 angiosperms. The major factor accounting for gene loss in the mitochondrial genomes is transfer to the nuclear genome. Presence or absence of mitochondrial genes in the nuclear genome have been reported for several species [45-48], including the transfer of *rps10*[49] and a lack of *sdh3* and *sdh4*[47] in the nuclear genome of carrot. In contrast with these data, our results revealed the presence of a nuclear copy of *sdh4* in the carrot genome. In addition, we confirmed the transfer of *rps10* from the mitochondria into the nucleus and detected an additional nuclear copy of *rps14*. We also found a replacement of the original mitochondrial *rpl2* with a copy of the plastid *rpl2* in the mitochondria. The high level of similarity between the *rpl2* nucleotide sequences in these two organellar genomes (99%) suggests a recent translocation. The lack of any sequence similar to the mitochondrial *rpl2* gene in the nuclear genome suggests that the original mitochondrial version has been lost. According to Kubo and Arimura [50], the replacement of a mitochondrial gene by a duplicated plastid counterpart could be due to the complete loss of the gene in either mitochondrial or nuclear genomes. However, detection of the ORFs for nuclear *sdh3rps10rps14* and plastid *rpl2* suggests the possibility of functionality. Further work will be needed to determine whether these nuclear copies are expressed.

The structure of angiosperm mitochondrial genomes is frequently characterized by repeat sequences [51]. The number and the size of these repeats is important, as they influence the size of the genome, and they are the sites of intragenomic recombination, underlining evolutionary changes in mitochondrial genome organization and structural dynamism *in vivo*[52,53]. A high proportion of smaller repeat sequences explained (<1 kb) the size of the largest mitochondrial genome of *Cucumis melo*[28]. In contrast, in maize [9], variable genome size among different genotypes is mainly caused by large (>1 kb) sequence duplication. The structure of the carrot mitochondrial genome presented here revealed that nearly half (46%) of the genome is composed of large repeated sequences. The fact that carrot has the least number of repeat sequences among sequenced mitochondrial genomes and the presence of four large repeats (1–14 kb), we deduced that the increase of the carrot mitochondrial genome size mainly occurred by large sequence duplication.

Intragenomic recombination is an active phenomenon in the mitochondrial genome of angiosperms [53,54]. Recombination frequency seems to depend on the size of the repeats. Large size direct repeats (>1 kb) are associated with high frequency of homologous recombination producing sub genomic molecules observed in other mitochondrial genomes [53,54]. Our Southern blot results provide evidence of recombination across a large repeat 4 (Figure 3), and the *in vivo* co-existence of sub-genomic circles. Smaller repeats, however, suggest very low recombination frequency, producing aberrant asymmetric recombinant molecules that are generally present at very low copy number and hard to detect [55,56]. In this study, we tested a possible recombination across short repeats using next generation sequencing data from Roche 454 and Illumina platforms and our results did not support any recombination events (data not shown). It was shown recently that mitochondrial intergenomic recombination required stretches of similarity longer than 500 nt and remained under control of a nuclear gene *msh1*. Inactivation of *msh1* lead to increased frequency of recombination and allowed recombination between repeats only 50 nt-long [57].

### DNA insertion in the plastid genome

Intercompartmental (plastid, mitochondrion and nucleus) DNA migration is a known phenomenon in plant cell evolution. DNA transfer among these compartments resulted in a functional relocation of organellar genes in the early phase of organelle evolution [13,56-60]. Theoretically, six intercompartmental DNA migrations are possible, and at least four have been observed in angiosperms (Additional file 9: Figure S7). Transfers of DNA from mitochondrion to nucleus and from plastid to mitochondrion are discussed above (see Introduction and Results section). DNA migration from plastid to nucleus has been described in *Arabidopsis,* soybean and other species [61,62]. Nuclear DNA has been transferred to the mitochondrial genome of *Cucumis melo*[28] and other species [10,63]. In contrast, no evidence of transfer of DNA from nucleus or mitochondria to plastid has been reported in angiosperms [13]. Smith [14] investigated the presence of mitochondrial DNA in the plastid genome of 42 species including 11 angiosperm, and found a complete absence of mtDNA like sequences in any of the plastid genome of these species. Recently Goremykin and colleagues [5] reported a fragment of mitochondrial DNA in *Vitis* with similarity to a sequence of 1452 nt present only in the carrot plastid genome.

In this study, we confirmed the presence of this sequence in the plastid genome of carrot, and discovered that it was also present as three non-contiguous sequences in the mitochondrial genome. PCR results and sequence analysis clearly document conservation of a large portion of this sequence (*Dc*MP region 3, Figure 5B) across all mitochondrial genomes of the diverse Apiaceae species examined, and a “gain pattern” only in *Daucus* and *Cuminum* plastid genomes. This evidence strongly suggests a transfer of DNA into the plastid genome. In addition, the observation of the insertion of the *Dc*MP region 4 in the plastid genome of wild *Daucus* species *D. aureusD. crinitus* and *D. glochidiatus* led us to hypothesize that in the ancestor of carrot the *Dc*MP regions 1, 2, 3 and 4 were contiguous when the transfer event occurred. Assuming a single transfer event, the loss of the sequence contiguity in the mitochondrial genome could be due to a recombination of that genome, as is often observed in plant mitochondrial genomes. According to Downie et al. [64], *Cuminum* is the closest relative to carrot of the taxa examined here. The PCR patterns obtained across species for the plastid copy of *Dc*MP concur with that classification and suggest that the presence of the *Dc*MP region can be a distinctive feature of species from the *Daucus* clade.

The fact that the D*c*MP3 sequence has no matches in sequence data bases makes interpretation of its origin difficult. Limited similarity with a retrotransposon element (in the range of 50–60%; Additional file 7: Figure S5) of *Vitis vinifera* and other species suggests that a retrotransposable element might have been moved from the nuclear genome into the mitochondrial and then into the plastid genome, or directly and independently from the nuclear genome to plastid and mitochondrial genomes. The fragment of 125 nt at the 5′ end of *Dc*MP3 is very conserved across several even more diverse angiosperm mitochondrial genomes, which suggests a mitochondrial origin, and also suggests that the transfer occurred from mitochondrial to plastid genome. However, considering the exceptional DNA up take system of plant mitochondrial genomes [4,65], other sources of DNA donors via horizontal transfer or transfer from the nuclear genome cannot be excluded. Rare insertions of retrotransposon elements into the plastid genome have been observed in the alga *Chlamydomonas reinhardtii*[66], but based on our knowledge this is the first evidence of transfer of DNA from mitochondrial genome to plastid genome, and the potential presence of a possible retrotransposon in the plastid genome of a flowering plant.

## Conclusions

Our results confirmed that unenriched whole genome sequencing is a practical approach for *de novo* assembly of higher plant mitochondrial genomes. Sequence analysis confirmed the dynamic organization of the mitochondrial genomes in carrot as in other mitochondrial genomes. This first report of a DNA insertion from the mitochondria in the angiosperm plastid genome suggests that new and unknown mechanisms in intercompartimental genome interactions may exist. The new approach developed here for assembly of the mitochondrial genome used here could be used more broadly to sequence and assemble mitochondrial genomes of diverse plant species. This would supplement the currently limited number of sequenced mitochondrial genomes, and may allow us to better understand intra- and inter-genomic DNA transfers and recombination, and this new instance of transfer of DNA to plastid genomes could possibly have occurred in other angiosperms.

## Methods

### Plant material and DNA extraction

A single male fertile plant of USDA carrot inbred line B493B [67] was used in this study (Additional file 10: Table S3). Leaf tissue was lyophilized, and extracted with DNeasy Plant Maxi Kit (Qiagen). The extracted DNA was analyzed for potential degradation by gel electrophoresis, and DNA concentration was quantified using Pico Green (Invitrogen, Pisley, UK).

DNA samples used for PCR verification of DNA transfer (*Coriandrum sativumPetroselium crispum* cv “Hamburg”, *Cuminum cyminumApium graveolens* ssp. *repaceum* cv ‘Brilliant’, *Anethum graveolensFoeniculum vulgareDaucus carota* ssp. *sativusDaucus muricatusDaucus glochidiatusDaucus crinitusDaucus capillifoliusDaucus aureusDaucus sahariensisDaucus pusillus*, and *Eryngium planum*) (Additional file 10) were extracted as described by Murray and Thompson [68] and quantified using a NanoDrop spectrophotometer (NanoDrop Technologies).

### Sequencing, assembly and finishing

454 sequencing was performed with a GS-FLX platform (Roche, CT, USA), and Illumina sequencing was performed with the HiSeq 2000 platform (Illumina, San Diego, CA). In both cases, the sequencing was done at the University of Wisconsin, Biotechnology Center (University of Wisconsin, Madison, USA). Shotgun libraries were prepared and sequenced according to the manufacturers’ instructions. For 454 sequences sets of 813,770, 814,668, 771,864, 704,918 and 692,688 shotgun reads corresponding to an estimated nuclear genome coverage of 0.6×/set were used for initial assembly and 570,590 3 kb paired-end reads were used for connections verification. In addition, 50,598,879 Illumina reads were used to correct homopolymer ambiguity.

Each set of 454 sequences was independently assembled using gsAssembler v.2.6 (Newbler) (454 Life Sciences Corp, CT, USA). Parameter settings are listed in additional file 11: Table S4. In order to identify contigs of plastid and mitochondrial origin, assembled sequences were aligned against the carrot plastid genome [GenBank: DQ898156] and tobacco mitochondrial genome [GenBank: NC006581] using MUMmer 3.22 [69]; http://mummer.sourceforge.net/. Contigs with similarity to *atpA* for plastid, or *atp1* for mitochondrion, were used as starting points for a *de novo* assembly of contigs. In order to visualize the contig connection information in the 454ContigGraph.txt file produced by gsAssembler, we created the program bb.454contignet (http://www.vcru.wisc.edu/simonlab/sdata/software/). Parameters are listed in additional file 11: Table S4. In order to generate a consensus sequence the five replicate assemblies, sequences were aligned using kalign [23] and a consensus generated using a custom Perl program.

To verify contig and repeat connections, sequences spanning those connections were amplified and sequenced using primer pair 1–12 listed in Additional file 11: Table S5. Fragments were amplified in a 20 μl PCR reaction: 13 μl water, 2 μl 10x DNA polymerase buffer, 0.8 μl dNTPs (2.5 mM each), 1 μl 5 μM of each primer, 0.2 μl *Taq* polymerase (MBI, Fermentas, USA) and 2 μl of genomic DNA (~50 ng). Amplification conditions were: initial denaturation at 94°C for 2 min., followed by 35 cycles of 94°C for 45 sec., Tm (°C) for 1.0 min., 72°C for 1.0 min. and 20 sec., and a final step at 72°C for 10.0 min. Electrophoresis was carried out for 2–3 hours at 100 V on 2% agarose TAE gels supplemented with 0.2 μg/ml of ethidium bromide. PCR products were then sequenced in a 5 μl reaction including, 1.75 μl of water, 1 μl of 5 μM primer, 0.75 μl 5× BigDye®3.1 sequencing buffer, 0.5 μl of BigDye®3.1 ready reaction mix and 1 μl of PCR product, previously diluted 1:10 with water. Amplification conditions were: 25 cycles of 96°C for 10 sec., and 58°C for 2 min., and a final step at 72°C for 5.0 min. The sequences were analyzed on an ABI 3730xl DNA Analyzer and analyzed using Sequencher software version 4.8 (GeneCodes Corporation, Ann Arbor, MI).

Sequences similar to plastid and mitochondrial genomes longer than 358 nt (which corresponds to the average read length) were amplified using primer pairs 13–18 reported in additional file 12: Table S5. PCR assay and sequence analysis were carried out as described above.

In addition, the consensus sequence was compared using blastn to a local database containing a set of 3 kb 454 paired-end reads and results were filtered and visualized with Circos (http://circos.ca/).

To correct for homopolymer length errors and evaluate genome coverage, both Illumina and 454 shotgun reads were mapped to the mitochondrial consensus sequence using GNUMAP v.3.0.0 [26], http://dna.cs.byu.edu/gnumap/. Homopolymer regions of ≥5nt were corrected by a custom Perl program when a majority of Illumina reads indicated a different length, in most cases of correction this became 1 nt longer.

### Southern blot hybridization

The DNA was digested in separate tubes with the Fermentas enzymes *Xho*I, *Eam*1105I (*Ahd*I) and *Ksp*AI (*Hpa*I). Each digestion was performed overnight in 37°C in 40 μl containing approx. 1 μg of DNA. After restriction the samples were supplemented with 8 μl of the 6X Loading Dye Solution (Fermentas) and run in a 0.7% agarose gel in TAE buffer, during the first hour at 0.9 V/cm and then for the next 15 h at 0.5 V/cm. After electrophoresis the gel was subjected to depurination, denaturation, neutralization and capillary blotting using procedures described in Roche DIG Application Manual. The probes were synthesized using long PCR carried out in 25 μl containing: 2.5 μl 10x Long PCR Buffer with MgCl_2_, 2.5 μl dNTPs (2.5 mM each) , 1 μl 1 μM of each primer, 0.2 μl of Long PCR Enzyme Mix (MBI, Fermentas, USA), and 2 μl (70 μM) of alkali-labile DIG-11-dUTP (Roche, Applied Science) and 2 μl of genomic DNA (~4 ng). Probes were synthesized using primer pairs 20–22 listed in additional file 12: Table S5 with the following PCR conditions: initial denaturation at 94°C for 2 min.; 10 cycles of 94°C for 20 sec., 57°C for 30 sec., 68°C for 15 min.; 25 cycles of 94°C for 20 sec., 57°C for 30 sec., 68°C for 15 min. with an automatic 10 sec. extension in each consecutive cycle and finally 10 min. in 68°C. Three μl of the labeling reaction were examined in the standard agarose gel against the unlabelled control (the reaction without DIG-11-dUTP). The remaining portion of the labeling reaction was added to 16 ml of the standard hybridization buffer, the resulting solution was incubated in a water bath at 95°C for 10 min. (probe denaturation) and afterwards immediately applied to the membrane of ca. 400 cm^2^.

### Sequence annotation

A preliminary annotation of proteins, rRNA, and tRNA was carried out using BLAST with a local database of nucleotide and protein sequences of all published mitochondrial genomes. Consistency of ORFs was then tested using Open Reading Frame Finder http://www.ncbi.nlm.nih.gov/gorf/gorf.html. The tRNA genes were detected with tRNAscan-SE [70], http://lowelab.ucsc.edu/tRNAscan-SE/. Any ORFs longer than 300 nt and not overlapping conserved genes were searched with the NCBI nucleotide and protein databases and annotated on the mitochondrial sequence. Sequence of *sdh3sdh4rpl2rps10* and *rps19* from the *Nicotiana tabacum* mitochondrial genome [GenBank: NC_006581] and *rps2rps14* from the *Vitis vinifera* mitochondrial genome [GenBank: NC_012119] were used to investigate the presence of those genes in the nuclear genome of carrot. In order to annotate sequences common to plastid and mitochondrial genomes, known conserved mitochondrial genes were first masked in our mitochondrial consensus sequence, which was then queried against a local database containing sequences of all published plastid genomes. Sequences with minimum length of 40 nt and 90% identity not interrupted by masked sequences were added to our annotation.

Regions repeated within the mitochondrial genome were detected with blastn with the following parameters: minimum length 40 nt; percent identity 90.

The complete sequence of the annotated carrot mitochondrial genome was deposited in the NCBI organelle genome database with accession number JQ248574.

### Verification of DNA insertion into plastid genome

Mitochondrial and plastid DNA insertions were amplified using primer pairs 15, 16 and 19 listed in additional file 12: Table S5. PCR assay and sequence analysis were carried out as described above. Sequences were then searched against the NCBI nucleotide and protein database using default parameters.

## Competing interests

The authors declare that they have no competing interests.

## Authors’ contributions

MI: designed experiments, carried out PCR validations and Sanger sequencing, annotated the genome, interpreted the data and wrote most sections of the manuscript; DSe: designed experiments, carried out most of the bioinformatic analyses, wrote relevant parts of the methods and revised the paper; MS: carried out Southern experiments, wrote the relative methods and revised the paper; DG: participated in data interpretation and revision of manuscript; DSp: provided taxonomic advice and reviewed the paper; PWS: developed the plant material, participated in the experimental design and initiation of the experiments, data interpretation, writing and revision of several sections of the manuscript. All authors read and approved the final manuscript.

## Supplementary Material

Additional file 1 **Figure S1.** Connections verification. **A**. Schematic representation of the order of single copy regions (A, B, C, D, E) and repeated regions (R1-R4) into two possible master circles, Mc 1 and Mc 2; **B**: PCR results of all possible region connections; letter above each lane indicate the location of the primer pair (relative to each region) used for PCR. MW: 1 kb DNA molecular weight; C- = negative control. Click here for file

Additional file 2 **Figure S2.** Comparison of the five mitochondrial genome assemblies from sequence sets 1–5. Different colors identify different contigs. Triangles indicate missing connections between contigs. Letters in the consensus sequence indicate single copy regions (A-E) and repeated regions (R1-R4). Click here for file

Additional file 3 **Table S1.** Summary of Illumina and 454 read coverage. Click here for file

Additional file 4 **Figure S3.** Coverage plots displaying the Illumina **(A)** and 454 **(B)** read coverage (Red line) and GC content (Green line) across the complete plastid **(A1, B1)** and mitochondrial **(A2, B2)** genomes. Windows in the graphs indicate regions with higher GC content. Click here for file

Additional file 5 **Figure S4.** Original Southern blot pictures for repeat 1-3-4. Unrelated samples should be ignored. Click here for file

Additional file 6 **Table S2.** Comparison of gene content in mitochondrial genomes. Click here for file

Additional file 7 **Figure S5.** Results of alignment of the *Dc*MP 3 sequence against the NCBI protein database. Click here for file

Additional file 8 **Figure S6. Potentiality of next generation sequencing data for assembly of plant mitochondrial genomes.** Number and size of repeat regions among representative sequenced plant mitochondrial genomes compared to the longest read length available for the two most utilized next generation sequencing technologies. Click here for file

Additional file 9 **Figure S7. Intercompartmental DNA transfer.** Schematic representation of all possible and observed directions of intercompartmental DNA transfer in angiosperm genomes. Black solid arrows indicate previously observed DNA transfers; black dotted arrow indicates unobserved DNA transfer; Red solid arrow indicates the mitochondrial-to-plastid transfer identified in carrot. Click here for file

Additional file 10 **Table S3.** Plant material used in this study. Click here for file

Additional file 11 **Table S4.** Parameters used for analysis programs. Click here for file

Additional file 12 **Tables S5.** Primers used in this study. Click here for file
