# Supplementary material for: De novo assembly of the carrot mitochondrial genome using next generation sequencing of whole genomic DNA provides first evidence of DNA transfer into an angiosperm plastid genome
Source: BMC Plant Biol. 2012 May 1;12:61. doi: 10.1186/1471-2229-12-61 (PMC3413510; doi:10.1186/1471-2229-12-61)
Supplement: Additional file 1 — Figure S1. Connections verification. A. Schematic representation of the order of single copy regions (A, B, C, D, E) and repeated regions (R1-R4) into two possible master circles, Mc 1 and Mc 2; B: PCR results of all possible region connections; letter above each lane indicate the location of the primer pair (relative to each region) used for PCR. MW: 1 kb DNA molecular weight; C- = negative control. [file 1471-2229-12-61-S1.pdf]

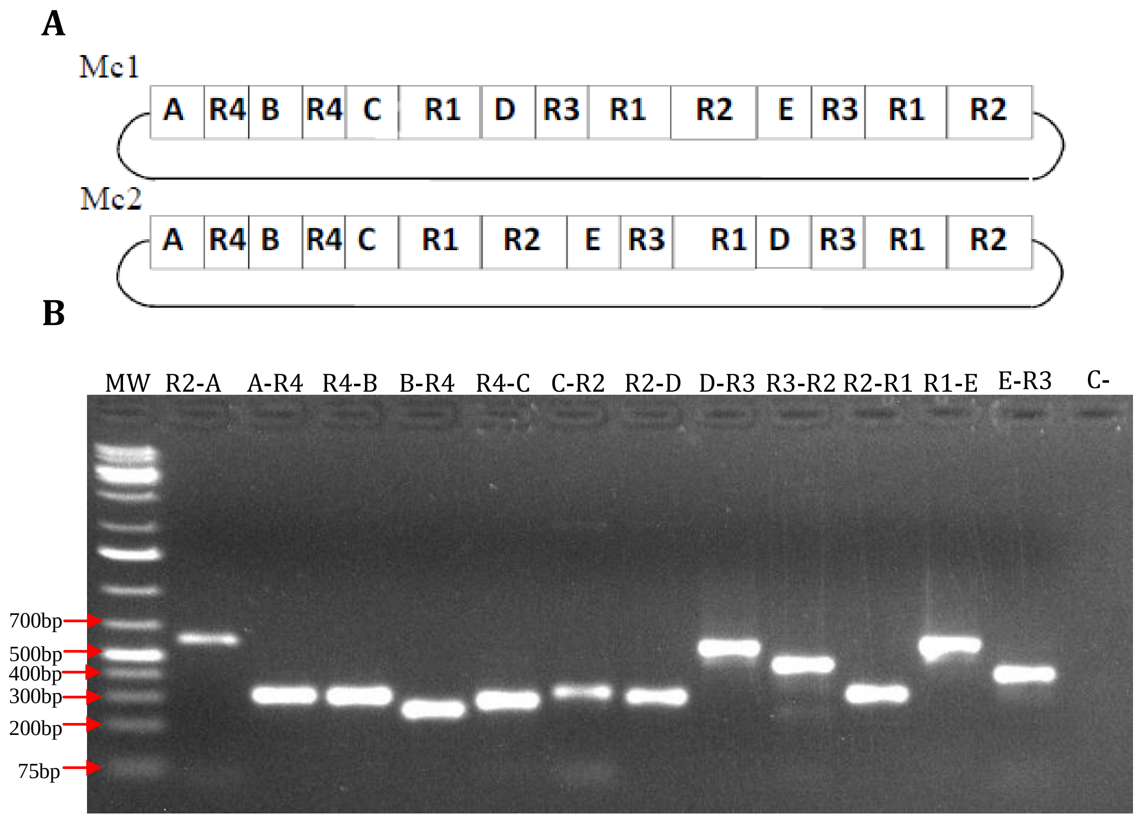

Figure S1. Connections verification. A: Schematic representation of the order of single copy regions (A, B, C, D, E) and repeated regions (R1-R4) into two possible master circles, Mc 1 and Mc 2; B: PCR results of all possible region connections; letter above each lane indicate the location of the primer pair (relative to each region) used for PCR. MW: 1 kb DNA molecular weight; C- = negative control.
