# Supplementary material for: De novo assembly of the carrot mitochondrial genome using next generation sequencing of whole genomic DNA provides first evidence of DNA transfer into an angiosperm plastid genome
Source: BMC Plant Biol. 2012 May 1;12:61. doi: 10.1186/1471-2229-12-61 (PMC3413510; doi:10.1186/1471-2229-12-61)
Supplement: Additional file 2 — Figure S2. Comparison of the five mitochondrial genome assemblies from sequence sets 1–5. Different colors identify different contigs. Triangles indicate missing connections between contigs. Letters in the consensus sequence indicate single copy regions (A-E) and repeated regions (R1-R4). [file 1471-2229-12-61-S2.pdf]

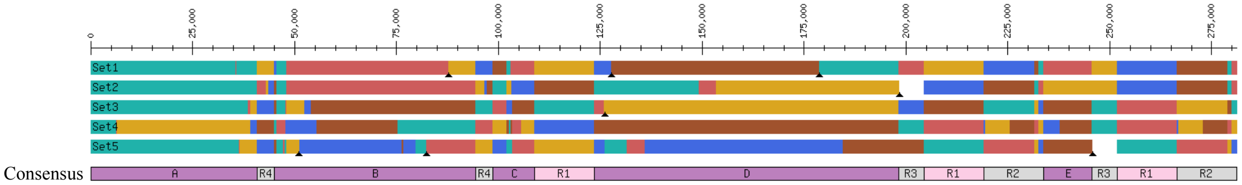

Figure S2. Comparison of the five mitochondrial genome assemblies from sequence sets 1-5. Different colors identify different contigs. Triangles indicate missing connections between contigs. Letters in the consensus sequence indicate single copy regions (A-E) and repeated regions (R1-R4).
