# Supplementary material for: De novo assembly of the carrot mitochondrial genome using next generation sequencing of whole genomic DNA provides first evidence of DNA transfer into an angiosperm plastid genome
Source: BMC Plant Biol. 2012 May 1;12:61. doi: 10.1186/1471-2229-12-61 (PMC3413510; doi:10.1186/1471-2229-12-61)
Supplement: Additional file 4 — Figure S3. Coverage plots displaying the Illumina (A) and 454 (B) read coverage (Red line) and GC content (Green line) across the complete plastid (A1, B1) and mitochondrial (A2, B2) genomes. Windows in the graphs indicate regions with higher GC content. [file 1471-2229-12-61-S4.pdf]

A1

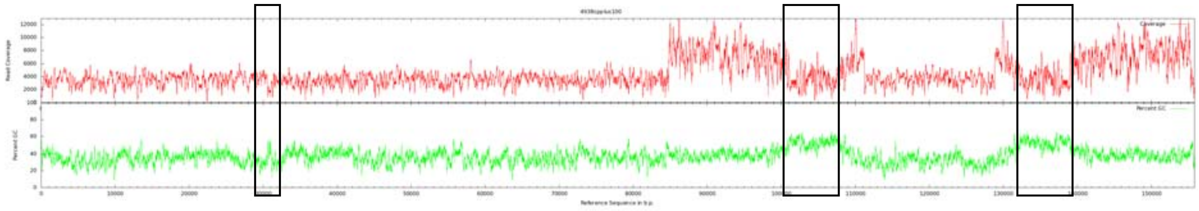

B1

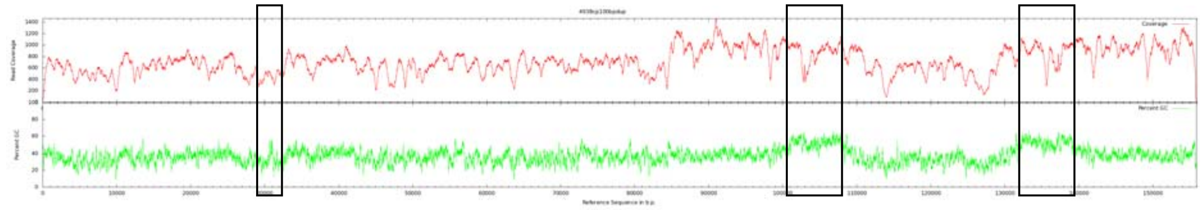

A2

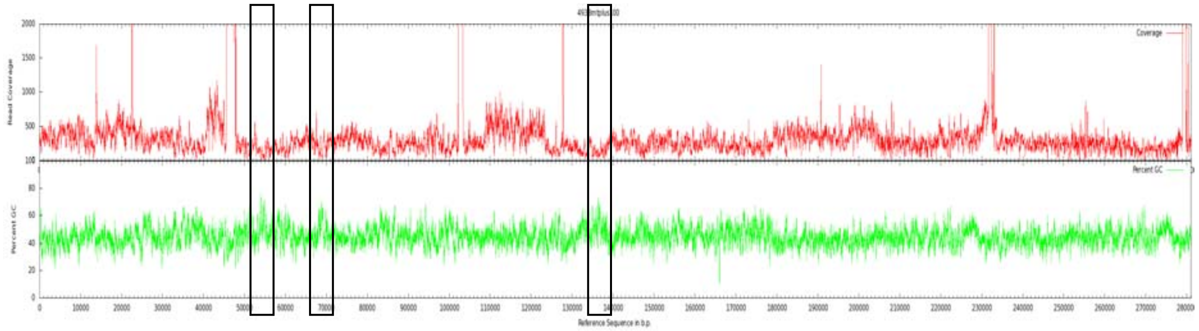

B2

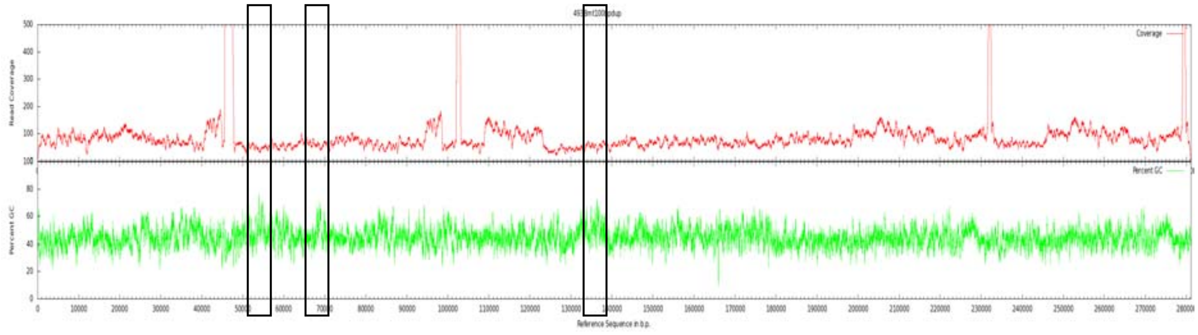

Figure S3. Representative line graph displaying the Illumina (A) and 454 (B) reads coverage (Red line) and GC contents (Green line) across the complete plastid genome (A1-B1) and mitochondrial (A2-B2). Windows in the graph indicate region with higher GC content.
