# Supplementary figures and images for: De novo assembly of the carrot mitochondrial genome using next generation sequencing of whole genomic DNA provides first evidence of DNA transfer into an angiosperm plastid genome
Source: BMC Plant Biol. 2012 May 1;12:61. doi: 10.1186/1471-2229-12-61 (PMC3413510; doi:10.1186/1471-2229-12-61)

**Figure S4.** Original Southern blot pictures for repeat 1-3-4.

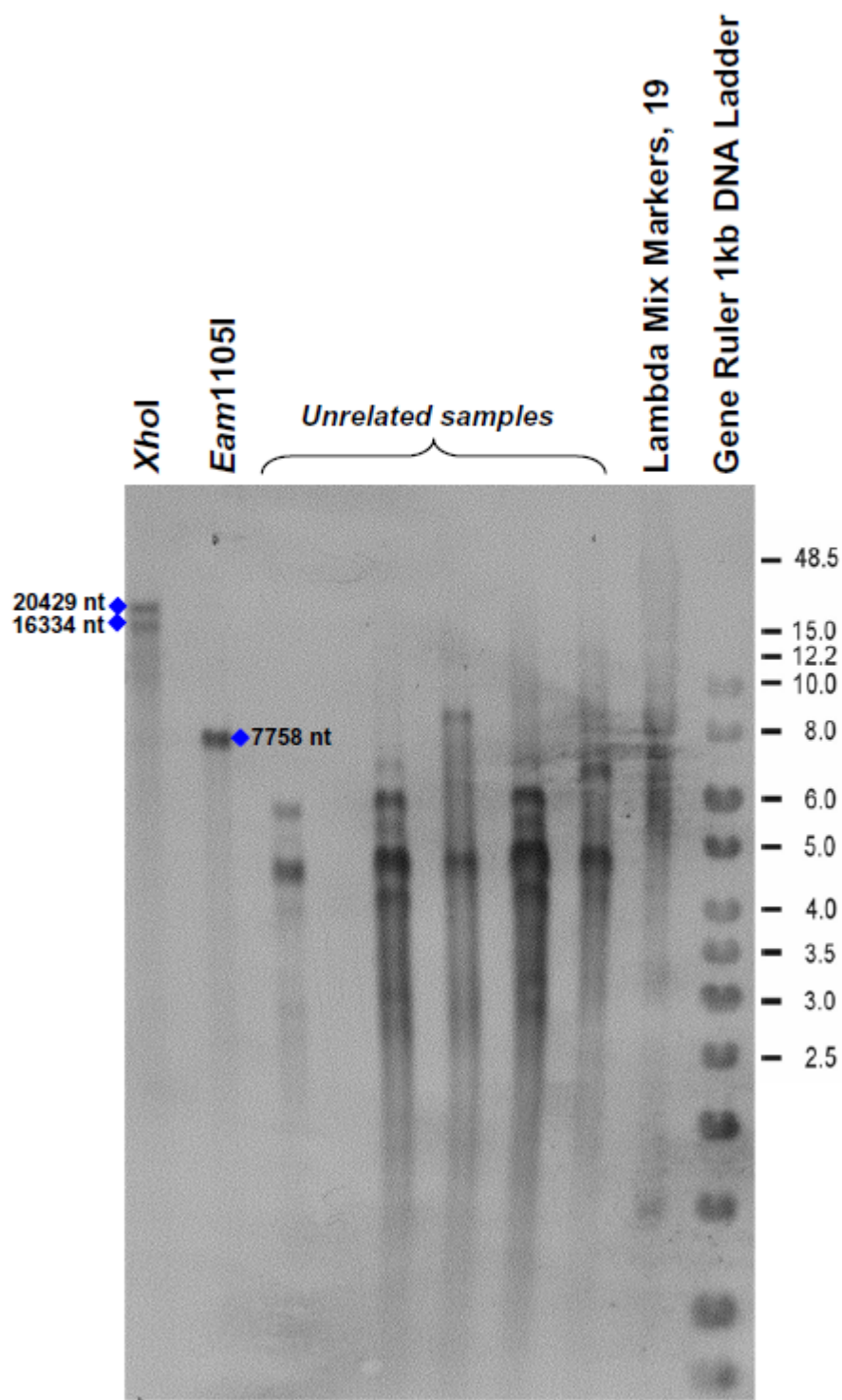

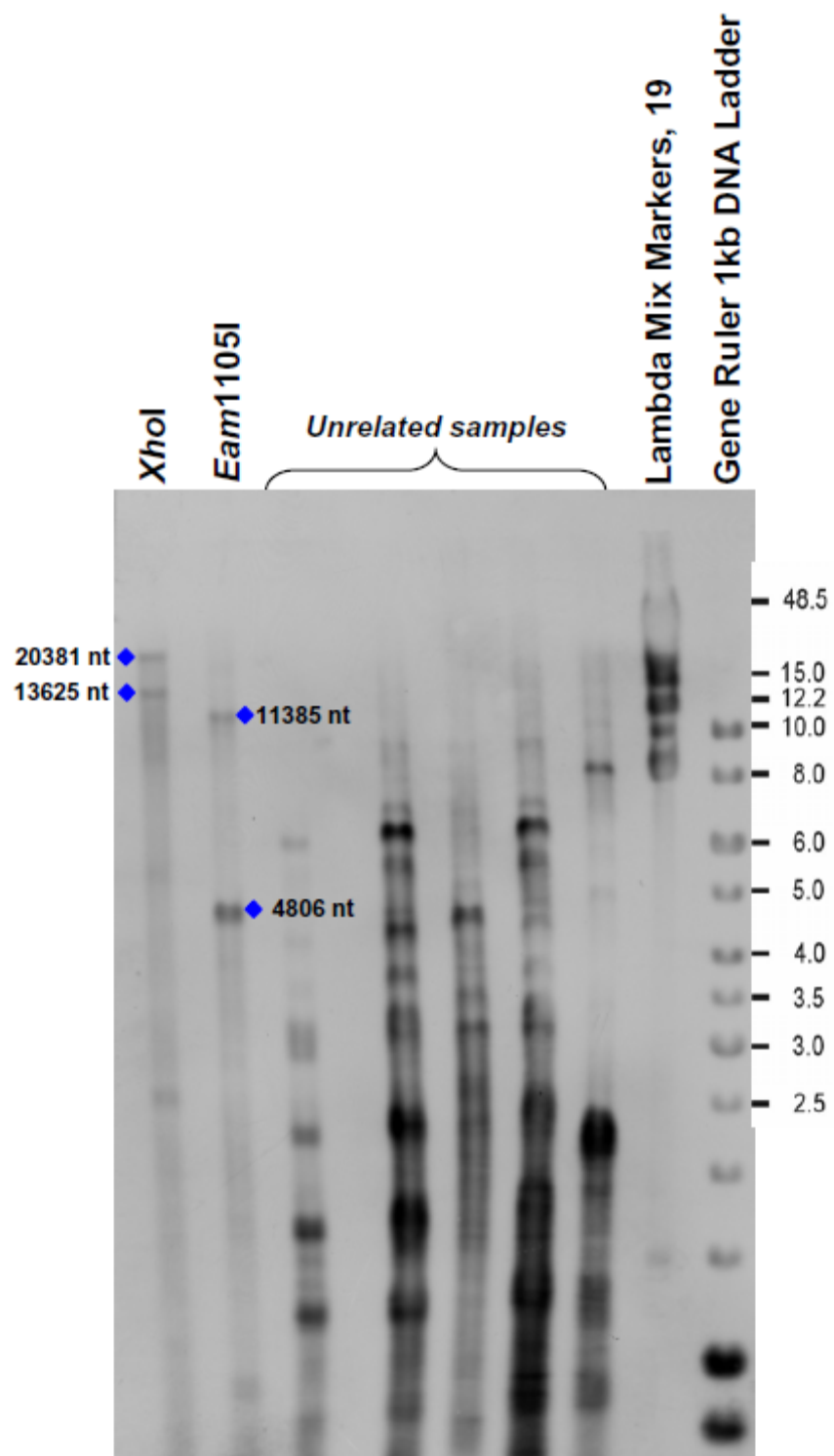

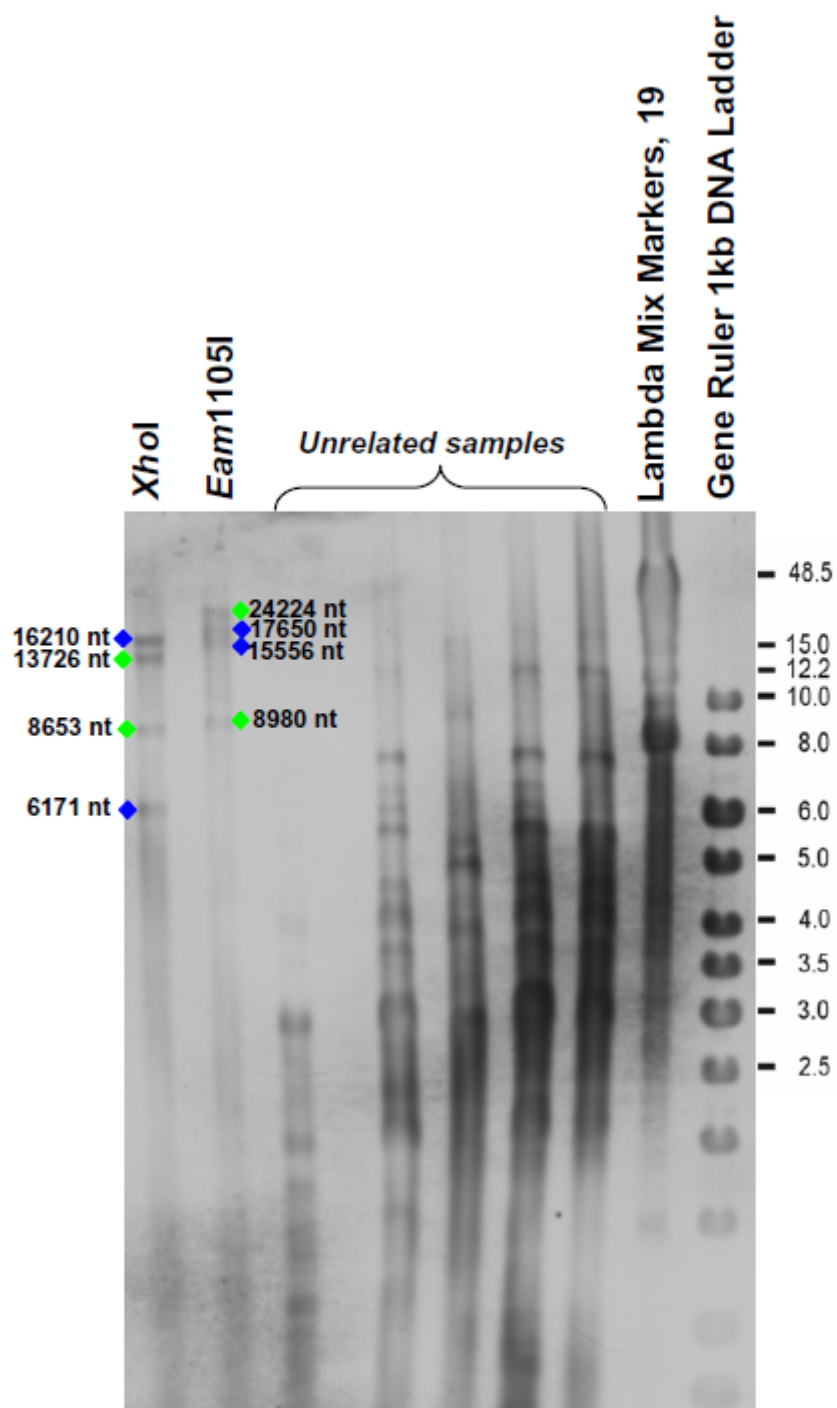

Supplement: Additional file 5 — Figure S4. Original Southern blot pictures for repeat 1-3-4. Unrelated samples should be ignored. [file 1471-2229-12-61-S5.pdf]

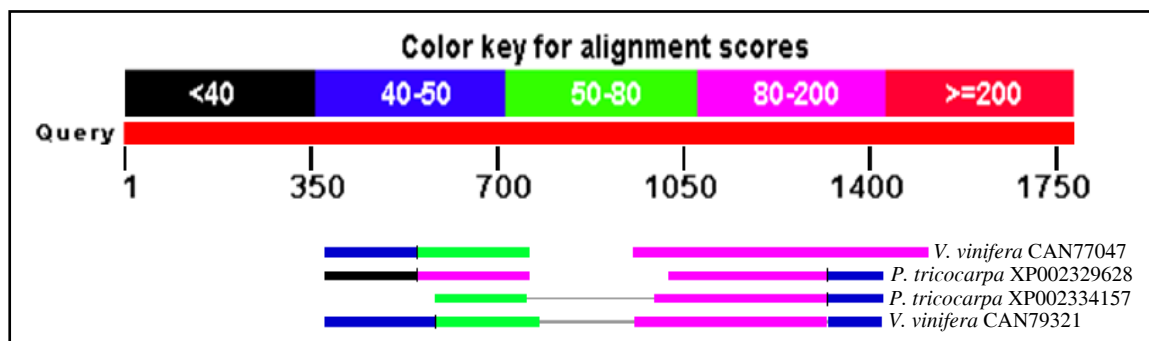

**Figure S5.** Results of alignment of the *DcMP 3* sequence against the NCBI protein database.

Supplement: Additional file 7 — Figure S5. Results of alignment of the DcMP 3 sequence against the NCBI protein database. [file 1471-2229-12-61-S7.pdf]
