# Supplementary material for: De novo assembly of the carrot mitochondrial genome using next generation sequencing of whole genomic DNA provides first evidence of DNA transfer into an angiosperm plastid genome
Source: BMC Plant Biol. 2012 May 1;12:61. doi: 10.1186/1471-2229-12-61 (PMC3413510; doi:10.1186/1471-2229-12-61)
Supplement: Additional file 8 — Figure S6. Potentiality of next generation sequencing data for assembly of plant mitochondrial genomes. Number and size of repeat regions among representative sequenced plant mitochondrial genomes compared to the longest read length available for the two most utilized next generation sequencing technologies. [file 1471-2229-12-61-S8.pdf]

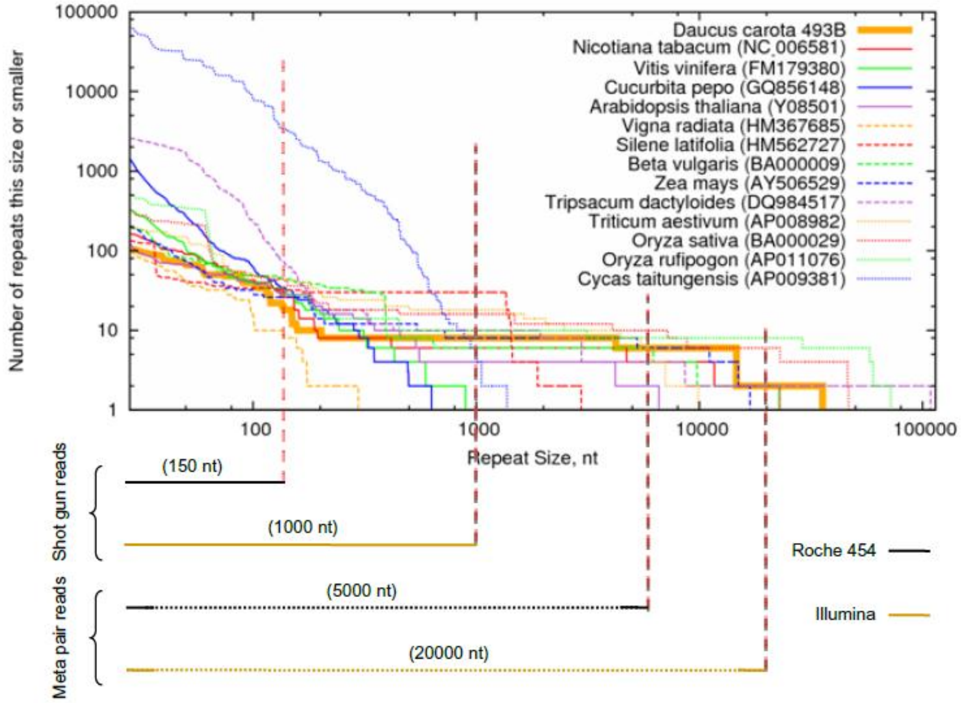

Figure S6. Potentiality of next generation sequencing data for assembly of plant mitochondrial genomes. Number and size of repeat regions among representative sequenced plant mitochondrial genomes compared to the longest read length available for the two most utilized next generation sequencing technologies.
