# Supplementary material for: De novo assembly of the carrot mitochondrial genome using next generation sequencing of whole genomic DNA provides first evidence of DNA transfer into an angiosperm plastid genome
Source: BMC Plant Biol. 2012 May 1;12:61. doi: 10.1186/1471-2229-12-61 (PMC3413510; doi:10.1186/1471-2229-12-61)
Supplement: Additional file 9 — Figure S7. Intercompartmental DNA transfer. Schematic representation of all possible and observed directions of intercompartmental DNA transfer in angiosperm genomes. Black solid arrows indicate previously observed DNA transfers; black dotted arrow indicates unobserved DNA transfer; Red solid arrow indicates the mitochondrial-to-plastid transfer identified in carrot. [file 1471-2229-12-61-S9.pdf]

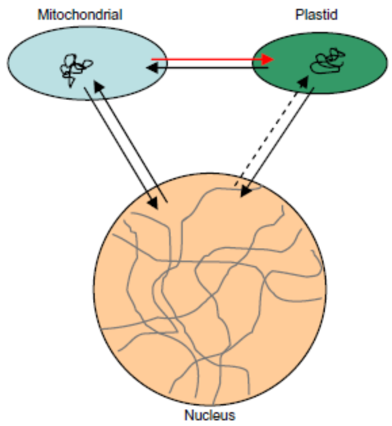

Figure S7. Intercompartmental DNA transfer. Schematic representation of all possible and observed directions of intercompartmental DNA transfer in angiosperm genomes. Black solid arrows indicate previously observed DNA transfers; black dotted arrow indicates unobserved DNA transfer; Red solid arrow indicates the mitochondrial-to-plastid transfer identified in carrot.
