# Supplementary material for: De novo assembly of the carrot mitochondrial genome using next generation sequencing of whole genomic DNA provides first evidence of DNA transfer into an angiosperm plastid genome
Source: BMC Plant Biol. 2012 May 1;12:61. doi: 10.1186/1471-2229-12-61 (PMC3413510; doi:10.1186/1471-2229-12-61)
Supplement: Additional file 11 — Table S4. Parameters used for analysis programs. [file 1471-2229-12-61-S11.pdf]

Table S4. Parameters used for analysis programs.

A. Parameters used for assembly of 454 sequence reads:

Software used:	Roche gsAssembler (Newbler)
Software version:	2.6 (20110517_1502)
Large or complex genome:	true
Heterozygotic mode:	false
Expected depth:	0 (=default)
Minimum read length:	20 (=default)
Parameters - Computation	
Number of CPUs to use (0=all):	14
Seed step:	12 (=default)
Seed length:	16 (=default)
Seed count:	1 (=default)
Minimum overlap length:	40 (=default)
Minimum overlap identity:	90 (=default)
Alignment identity score:	2 (=default)
Alignment difference score:	-3 (=default)
Parameters - Output	
Reads limited to one contig:	false
Pairwise alignment:	None
Alignment info:	True
All contig threshold:	1 (default=100)
Large contig threshold:	2,000 (default=500)

B. Parameters used for assembly of contigs

Software used:	bb.454contignet URL: http://www.vcru.wisc.edu/simonlab/sdata/software/index.html#contignet
Software version:	1.0.4
Parameters:	--len=3 --level=8 --extend=80
